# Anxiety and Stress Levels Associated With COVID-19 Pandemic of University Students in Turkey: A Year After the Pandemic

**DOI:** 10.3389/fpsyt.2021.731348

**Published:** 2021-10-29

**Authors:** Atahan Durbas, Hüseyin Karaman, Caǧla Hamide Solman, Nisanur Kaygisiz, Özdal Ersoy

**Affiliations:** ^1^School of Medicine, Acibadem Mehmet Ali Aydinlar University, Istanbul, Turkey; ^2^Department of Gastroenterology, Acibadem International Hospital, Istanbul, Turkey

**Keywords:** COVID-19, coronavirus, pandemic, anxiety, stress, university, students, young adults

## Abstract

The outbreak of COVID-19 has been affecting the daily lives of almost everyone and puts huge psychological pressure on people worldwide, including Turkey. Anxiety and stress levels among university students were already a public health concern. Our study aims to demonstrate the anxiety and stress levels of university students in Turkey after the outbreak of COVID-19 according to the Coronavirus Anxiety Scale (CAS) and COVID Stress Scale (CSS). CAS is a brief mental health screener to identify probable cases of dysfunctional anxiety associated with the COVID-19 pandemic, and CSS was developed to understand and assess COVID-19-related distress. An online questionnaire was administered to active 1,265 university students in Turkey between February 27 and March 8, 2021, *via* Google forms. The questionnaire consists of three parts that assess participants' demographic information, anxiety, and stress levels related to the pandemic. According to CAS and CSS analysis, anxiety and stress levels were associated with each other and influenced university students during the COVID-19 pandemic. Both were associated with gender and family member loss. The academic year of students had a relationship with anxiety. It was observed that the danger factor was the highest stressor in university students in Turkey related to the novel coronavirus, followed by contamination fears. Both factors were shown as moderate stressors. As a result of the study, it was revealed that anxiety and stress associated with the COVID-19 pandemic are now included in the social, academic, and physical burdens of the university years, which are decisive and important in terms of mental development and psychological health of the person. It is essential to ascertain the long-term effects of COVID-19 and take effective precautions to support the physical and mental health of today's university students accordingly.

## Introduction

SARS-CoV-2, now called COVID-19 after its appearance in Wuhan, China in December 2019, is a novel member of the coronavirus family (1). The outbreak of COVID-19 has been affecting the daily lives of almost everyone, especially after the declaration of a global pandemic by the World Health Organization (WHO) in mid-March 2020 (2). The first case of COVID-19 in Turkey was announced on March 10, 2020 (3), and the first death occurred on March 17, 2020 (4). In response to the COVID-19 pandemic, the Turkish Government took a series of preventions to slow the spreading of the disease down, and pausing the countrywide higher education was one of them. Educational activities in all universities were suspended on March 18, 2020 (5). On March 26, 2020, the Turkish Higher Education Council decided that education in universities would be carried out *via* distance and digital learning, i.e., no in-person instruction was to happen (6). On August 13, 2020, the Turkish Higher Education Council announced that education in universities would continue to carry out mainly online after October 1 (7). On December 30–31, 2020, 3 million doses of vaccine that came to Turkey were received and the 14-day safety trials of the Turkish Medicines and Medical Devices Agency (TITCK) began (8). Then, the vaccination schedule was announced, and healthcare workers and older people started to be vaccinated. While the vaccination schedule continued, the controlled normalization process started on March 1, 2021. Although approximately 9 million people were vaccinated, the number of daily cases continued to increase day by day in March (9).

The evolution of COVID-19 has placed enormous stress on healthcare, economic, and social systems in Turkey, as well as in the world (10, 11). In addition to the risk of infection and possible death, the pandemic put huge psychological pressure on people worldwide (12–15). Several studies have discussed the short- and long-term effects of the pandemic on the social and psychological health of the world (15–17). These side effects may depend on the mental health outcomes for people who get infected directly by COVID-19 (18) or be related to preventions (19) and the socio-economic impacts (10).

Anxiety and stress levels among university students are already a public health concern. Uncertainty and worries because of the pandemic made the mental health of the university students worse. Many studies have been added to the literature investigating the relationship between university students and their stress and anxiety levels during COVID-19. Studies investigating stress and anxiety levels have been conducted among university students in China, France, Poland, Bangladesh (20–23), Saudi Arabia (24), and Jordan (25). Also, stress and anxiety levels of medical and non-medical students have been examined in United Arab Emirates (26) and Iran (27). In Pakistan, a study investigated the anxiety and depression of healthcare professionals, medical students, and the general population during the COVID-19 pandemic (28). According to the number of total and new COVID-19 cases founded, Turkey is one of the top 10 countries in the world (29); however, there are only a few numbers of studies emphasizing this connection in the literature (30–33). Furthermore, these studies have been conducted with a limited number of participants (34) and within very specific student groups (such as only medical students included) (32, 33) compared to similar studies in the world (20–28).

Our study aims to demonstrate the significant relationship between the anxiety and stress levels of university students in Turkey and the outbreak of COVID-19 according to the Coronavirus Anxiety Scale (CAS) (34) and the COVID Stress Scale (CSS) (35). Our study also aims to follow up on anxiety and stress levels after the first year of the pandemic. We hypothesize that anxiety and stress levels of university students in Turkey would be associated with the effect of the pandemic directly. According to the possible results, we can reflect the anxiety and stress levels of the Turkish university students in the most realistic way possible; thus, precautions to minimize their anxiety can be taken early and effectively for the future.

## Materials and Methods

### Ethical Considerations

This study was approved by the T.C. Ministry of Health (on February 21, 2021) and the Acibadem University Medical Research Ethics Committee (Protocol Code: ATADEK 2021 04/27). On the first page of the questionnaire, there is informed consent to the participants explaining the purpose of the study and anonymity of their responses.

### Study Design and Participants

Our study was conducted between February 27 and March 8, 2021. The questionnaire created *via* Google forms was used as the data collection tool for the study. Participants should be actively enrolled in Turkish universities. Not giving informed consent, not having a university education in Turkey, or having a break from university education are exclusion criteria. The questionnaire link was sent to participants *via* social media tools. Also, participants were asked to send the questionnaire link to other acquaintance university students to reach more participants from different universities. A total of 1,265 university students from 119 different universities located in 44 different cities in Turkey have participated in the study. The questionnaire was in the Turkish language; it consists of three parts and a total of 62 questions. Participants had to answer all the questions at the second and the third part of the questionnaire to submit the form and therefore to be included in the study. Moreover, participants who completed the second and the third part of the questionnaire but did not answer particular questions about background information were excluded for only the questions that they did not answer.

### Questionnaire

#### Demographic/Background Information

The first part asked about the participants' gender, age, which university, faculty, and the year they are enrolled in, their smoking and drinking habits, their psychological and psychiatric well-being, any prescribed drugs they are using, where they were living before the pandemic, and where they are living now.

#### Stress Scale

In the study, the Turkish version of the CSS (36) developed by Steven Taylor et al. consisting of 36 questions on five dimensions (danger and contamination, economic consequences, xenophobia, traumatic stress symptoms, and compulsive checking) was used to evaluate the stress levels of the participants. The question was rated on a five-point scale ranging from 1 (Not at all) to 5 (Extremely). The sum of the scores for 36 items ranges from 0 to 144. Total scores of 0–47 are considered as low stress level, 48–96 as medium stress level, and 97–144 as high stress level.

#### Anxiety Scale

In the study, the Turkish version of the CAS was used to determine the participants' anxiety levels. It is developed by Lee S.A. and adapted to Turkish by Biçer et al. (37). The scale consists of five items in total. Participants were asked to reply to each question, which is scored on a five-point Likert-type scale (1 = Not at all, 2 = Rare, less than a day or two, 3 = Several days, 4 = More than 7 days, 5 = Nearly every day over the last 2 weeks). The sum of the scores for five items ranges from 0 to 25. Total scores of 0 to 9 are considered as normal and scores of 10–25 are considered as anxiety.

### Statistical Analysis

The data were analyzed using Microsoft Excel (2019). The fitness of the distributions of the variables to the normal distribution was tested with the Shapiro–Wilk Test. *P* < 0.05 was chosen to demonstrate statistical significance. Numbers, percentages, mean, and standard deviation were used as the descriptive statistics for the population. Categorical variables were compared by using the chi-squared test. The multivariate regression analysis was utilized to determine the effects of attributable demographic factors on anxiety and stress.

## Results

A total of 1,265 students, from 119 universities, which were from 44 different cities of Turkey, participated in the online survey. A total of 245 students were excluded because they did not meet inclusion criteria. Of the total participants, 646 (63.34%) were female and 372 (36.47%) were male. Their ages were between 16 and 38 (21.06 ± 2.52). The rest of the demographics of the participants are presented in Table 1.

**Table 1:** Demographic information of participants.

	**Number of students** [*n* (%)]^*^
Gender	
Male	372 (36.5%)
Female	646 (63.3%)
Age (years)	
<18	6 (0.6%)
18–21	617 (61.2%)
22–25	355 (35.2%)
26–29	23 (2.3%)
> 29	7 (0.7%)
Smoking	
Yes	153 (15%)
No	867 (85%)
Alcohol Drink	
Regularly	28 (2.7%)
Socially	389 (38.1%)
Never	599 (58.7%)
Chronic Disease	
Yes	86 (8.4%)
No	925 (90.7%)
Prescription Drugs	
Yes	158 (15.5%)
No	856 (84%)
Mental Disorder	
Yes	224 (22%)
No	791 (77.5%)
COVID-19 Contact	
Yes	630 (61.8%)
No	382 (37.5%)

* *The total number of valid participants is 1,020. The difference between the total number of participants and the actual number of participants in each subgroup in the table is equal to the number of participants who did not answer that question*.

According to CAS analysis, 139 (13.63%) participants had anxiety related to the COVID-19 pandemic. However, the rest of them (86.37%) did not. Besides, participants' stress levels were also assessed according to CSS. CSS was a five-point Likert scale, and the results were divided into three groups as “low,” “medium,” and “high” for interpretation. Although 78 participants (7.65%) were classed as “low,” 406 (39.80%) of them had “high” stress levels. Nearly half of them (52.55%) showed “medium” level stress related to the COVID-19 pandemic. Furthermore, there was a strong association [$\chi_{\left(2,\;N=1,020\right)}^{2}$ = 275.38, *p* < 0.001] between having anxiety and stress level (Table 2).

**Table 2:** Results of chi-square tests of anxiety and stress.

**Outcomes**	**Anxiety (+)**	**Anxiety (–)**	**Total**
High stress	56	22	78
Moderate stress	77	459	536
Low stress	5	400	406
Total	139	881	1,020
	χ^2^ = 275.38		*P* < 0.001

The relationship between demographic features of participants and having COVID-19-related anxiety was investigated. The gender (female/male) of participants was strongly associated with COVID-19-related anxiety [$\chi_{\left(1,\;N=1,018\right)}^{2}$ = 12.69, *p* < 0.001] (Table 3). Additionally, students who lost a family member due to COVID-19 were significantly associated with having COVID-19-related anxiety [$\chi_{\left(1,\;N=1,012\right)}^{2}$ = 11.09, *p* < 0.001] (Table 4). Furthermore, COVID-19-related anxiety has a significant association with the participants' year of study [$\chi_{\left(2,\;N=1,012\right)}^{2}$ = 9.05, *p* = 0.01] (Table 5). No significant association between COVID-19-related anxiety and remaining demographic parameters was determined.

**Table 3:** Results of chi-square tests of gender and anxiety.

**Participants**	**Women**	**Men**	**Total**
Had COVID-19-related anxiety	107	32	139
Had no COVID-19-related anxiety	539	340	879
Total	646	372	1,018
	χ^2^ = 12.69		*p* < 0.001

**Table 4:** Results of chi-square tests of had lost a family member due to COVID-19 and anxiety.

**Participants**	**Had lost a** **family member** **due COVID-19**	**Had not lost** **a family member** **due COVID-19**	**Total**
Had COVID-19-related anxiety	106	33	139
Had no COVID-19-related anxiety	538	335	873
Total	630	382	1,012
	χ^2^ = 11.09		*p* < 0.001

**Table 5:** Results of chi-square tests of the academic year of students and anxiety.

**Participants**	**First grade students**	**Intermediate grade students**	**Final grade students**	**Total**
Had COVID-19-related anxiety	59	51	28	138
Had no COVID-19-related anxiety	311	274	289	874
Total	370	325	317	1,012
		χ^2^ = 9.05		*p* = 0.01

The association between COVID-19-related stress levels and the factors that may contribute to this is also investigated. The gender (female/male) of participants was found to be associated with stress levels [$\chi_{\left(2,\;N=1,018\right)}^{2}$ =28.42, *p* < 0.001] (Table 6). Besides, loss of a relative due to COVID-19 infection reveals an association with COVID-19-related stress levels [$\chi_{\left(2,\;N=1,016\right)}^{2}$ =14.68, *p* < 0.001] (Table 7). Use of cigarettes and alcohol, the presence of chronic illnesses and psychological disorders, and regularly taking pills are factors that have not been found to be significantly associated with COVID-19-related stress levels.

**Table 6:** Results of chi-square tests of gender and stress levels.

**Participants**	**Women**	**Men**	**Total**
High stress	56	22	78
Moderate stress	373	162	535
Low stress	217	188	405
Total	646	372	1,018
	χ^2^ = 28.42		*p* < 0.001

**Table 7:** Results of chi-square tests of had lost a family member due to COVID-19 and stress levels.

**Participants**	**Had lost a family member due COVID-19**	**Had not lost a family member due COVID-19**	**Total**
High stress	26	52	78
Moderate stress	89	444	533
Low stress	53	352	405
Total	168	848	1,016
	χ^2^ = 14.68		*p* < 0.001

The factors that have determined exploratory in the CSS scale are investigated (Table 8). Six exploratory factors questioned in the scale were; danger and contamination fears of COVID-19, COVID-19 fears about economic consequences, COVID xenophobia, COVID compulsive checking and reassurance-seeking, and COVID traumatic stress symptoms. The average points of the participants and the standard deviations of each relevant question to factors are assessed. COVID-19 danger fears was found to be the highest (*M* = 3.35, *SD* = 0.94), followed by contamination fears (*M* = 3.26, *SD* = 1.12), xenophobia related to COVID-19 (*M* = 2.66, *SD* = 1.16), compulsive checking and reassurance (*M* = 2.60, *SD* = 0.93), traumatic stress (*M* = 1.87, *SD* = 0.89), and fears of socioeconomic consequences (*M* = 1.80, *SD* = 0.92).

**Table 8:** Mean and standard deviations of different exploratory factors of CSS.

**Types of** **exploratory factors**	**Mean**	**Standard deviation**
Danger	3.35	0.94
Contamination fears	3.26	1.12
Xenophobia	2.66	1.16
Compulsive checking and reassurance	2.60	0.93
Traumatic stress	1.87	0.89
Fears of socioeconomic consequences	1.80	0.92

## Discussion

Our study indicated that anxiety and stress are related to each other. People who have higher anxiety levels are also having higher stress levels on the scales. This finding is compatible with previous studies (38–40). In addition, anxiety and stress levels are dependent on gender. We found that the points of anxiety and stress are significantly higher in women than in men. This presents that the psychiatric burden of the COVID-19 pandemic may be greater in particular groups. In our study, we found that anxiety prevalence Rates in males and females are 8.60 and 16.56%, respectively. These rates are relatively higher than other types of anxiety prevalence in former studies (41). This is expected as lockdowns, social and economic restrictions, and rapid changes to online implementations might be regarded as significant stressors for university students. COVID-19 is a traumatic factor that threatens people and their beloved relatives' lives and therefore affects community health physically and emotionally. Besides, females are found with significantly higher COVID-19 prevalence rates. Also in literature, women persistently exhibited higher prevalence rates, as high as two times, for other types of anxiety disorders (42, 43).

In our study, we showed that anxiety and stress levels are associated with losing a family member due to COVID-19. Students who had lost a family member probably are feeling depressed due to their loss and concerned that they have to experience the same situation again, which may lead to anxiety and stress (30, 32). Additionally, there is an association between anxiety and the year of study of the participants. This relationship is probably due to the uncertainty created by the pandemic. Younger students are in a new environment and do not know how long this condition will last exactly. On the other hand, older students may worry about their post-graduate work problems. Moreover, students are not on campuses, and they are taking their classes online. Online classes are a new approach for all students in Turkey and this might also cause anxiety in students (32, 39, 44, 45).

Unlike previous studies conducted in our country, we did not find any significant relationship between anxiety/stress levels and other potential factors such as smoking, alcohol, chronic disease, psychiatric problem, and clinical internship (30, 32). The high density of young participants in this study may be the reason for the difference with previous studies about COVID-19. The fact that young people will catch the disease relatively less and experience a milder illness may explain the unrelated situation between COVID-19-related anxiety/stress and attributable risk factors compared to the general population (46). Our study was conducted nearly 1 year after the pandemic, and thus, this fact may influence participants. Students are more aware of the pandemic and its risk factors and have more mental problems primarily due to limitations and restrictions in the country.

University students are a risk group for mental health disorders with high rates of psychiatric morbidity, primarily depression, and anxiety (47). Prevention and reduction of mental problems in university students are essential to support community health because this period is regarded as a sensitive period of a person's life in terms of psychosocial development (48). Research shows that already before the pandemic, undergraduate students show moderate to moderate-high stress (45, 49). In our study, we examine COVID-19-related stress levels using CSS. Low to moderate level stress for different exploratory factors related to coronavirus is shown. The danger factor is found to be the highest, which questioned worries about catching the virus and getting an infection for the person and beloved ones. Contamination fears come second in the order, which is indeed a subtype of obsessive–compulsive symptoms. Recent studies indicated a significant worsening in the severity of obsessive–compulsive disorders especially in terms of contamination (50). Our findings exhibit moderate levels of contamination fears, supposing obsessive–compulsive disorder incidence may have surged during the pandemic period. Together with the pandemic-related issues like social distancing and isolation, which provoked negative senses such as worry, anger, loneliness, and helplessness, xenophobic attitudes pose a danger to individual lives that even lead to suicide cases (51). COVID-19 was expected to negatively impact the global economy as international trade networks are disrupted. Furthermore, consumers stay at home longer, which results in a decrease in their consumption of services and goods and increases in their savings. While social distancing and lockdown measures affect a person's socioeconomic status such as impact labor status and financial income, mental health and well-being, and environmental factors (52–54), in this study, possible socioeconomic consequences and shortages are shown to be the last concern (avg. 1.80 [low-level stress]) of the students. The reason for this may be that besides the risk of losing one's own life or a loved one, the economic threats of COVID-19 seem to be relatively more tolerable.

In the study Akdeniz et al. (30) conducted about a year before our research, they stated that the anxiety levels of their participants were high in the first days of the pandemic, especially about losing a relative due to the pandemic in the future. As they discussed, it was a matter of interest how the concerns created by the atmosphere of uncertainty in those days would be shaped in the future. In our study a year later and in a similar population, it was shown that although the effects of the pandemic were better understood and settled on a more stable basis, the anxiety was still a concern. Besides, Fawaz et al. (55) investigated the relationship between rapid change to online learning implications and the prevalence of depression, anxiety, and stress, and they revealed that this change has negative effects on the mental health of university students. Our results also support these interpretations.

## Strengths and Limitations

The multi-centered structure of this study is one of its strongest points. Students participated from different years (ages) and majors, from more than half of the total provinces in Turkey; this could successfully reflect university students of Turkey in terms of anxiety and stress levels related to COVID-19. During the distribution process of the questionnaire, great attention and effort were paid to the participation of students from many cities, universities, and different faculties throughout Turkey, and as a result, the opportunity to analyze the results in a wide population and geography emerged. However, the fact that the study had a non-random design and that the distribution of the questionnaire was made by online social platforms may have caused some biases. First, students who had reached the survey link and filled out the survey may have different characteristics than those who do not. As a concrete manifestation of this situation, it can be put forward that women participate in the study more than men. Moreover, students who are better planned academically and with a high interest in extracurricular academic activities may have participated more in the study. Secondly, the fact that the answers of the participants completely reflected their own personal expressions, i.e., answers are self-reported, may have caused the distortion of some outcomes; for example, the deliberate incorrect expression of the participants using psychiatric medications may deliberately express incorrectly their usage status due to fear of social stigmatization. Measurement errors related to self-reported outcomes should be limited as the names of participants were not demanded, and questions were answered privately, and seen and analyzed by only researchers. In addition, the study may have been insufficient to reveal causality and the designed and analyzed correlation and the subject did not comply with controlled experimental studies in terms of ethics and structural difficulties. For example, it is unclear whether the fear of contamination, which is found at a moderate level in the study, is affected by COVID-19, or whether individuals who already have obsessive predispositions express their disorder with COVID-19.

In conjunction with the fact that this study started 1 year after the beginning of the pandemic in our country, the vaccinated part of the population was growing/expanding day by day, which might not directly reflect the effects on the first days of COVID-19 that is more panicky and ambiguous. This situation can explain the difference in our study compared to others that were conducted in the first days of the pandemic. However, our study reveals important outputs in terms of exhibiting the differences between the studies at the beginning of the pandemic and in the intervening year. The most important factors affecting the stress and anxiety levels in the students in this process are the new approaches in the treatment of COVID-19 and the development of vaccine technologies.

## Conclusion

In summary, anxiety and stress levels are related to each other and are high in university students during the COVID-19 pandemic. Both are associated with gender and family member loss. The year of study of the students also has a relationship with anxiety. It was observed that the danger factor is the highest stressor in university students in Turkey related to catching the novel coronavirus, followed by the contamination fears. Both factors are shown as moderate stressors. The factor at the lowest level in the stress scale measurements was determined as the socioeconomic factor. As a result of the study, it was revealed that in addition to the social, academic, and physical burdens of the university years, which are decisive and important in terms of the mental development and psychological health of the person, COVID-19 was also incorporated at the present conditions. Maintaining the physical and mental health of today's university students is critical to creating a healthy community structure after COVID-19 (post-COVID era). Therefore, it is important for public health, especially for young adults, to carry out ongoing studies that will continue to monitor the long-term effects of COVID-19, to provide psychological support to university students, and to shape education channels accordingly.

## Data Availability Statement

The raw data supporting the conclusions of this article will be made available by the authors, without undue reservation.

## Ethics Statement

The studies involving human participants were reviewed and approved by Acibadem Mehmet Ali Aydinlar University Medical Research Review Board (ATADEK). The patients/participants provided their written informed consent to participate in this study. Written informed consent was obtained from the individual(s) for the publication of any potentially identifiable images or data included in this article.

## Author Contributions

AD, HK, CS, and NK designed the study and wrote and revised the manuscript. ÖE supervised the study. HK, CS, and NK organized the database and resources. AD and HK conceived the statistical approach and performed the statistical analysis. All authors approved the submitted version.

## Conflict of Interest

The authors declare that the research was conducted in the absence of any commercial or financial relationships that could be construed as a potential conflict of interest.

## Publisher's Note

All claims expressed in this article are solely those of the authors and do not necessarily represent those of their affiliated organizations, or those of the publisher, the editors and the reviewers. Any product that may be evaluated in this article, or claim that may be made by its manufacturer, is not guaranteed or endorsed by the publisher.
