# Supplementary material for: Anxiety and Stress Levels Associated With COVID-19 Pandemic of University Students in Turkey: A Year After the Pandemic
Source: Front Psychiatry. 2021 Oct 29;12:731348. doi: 10.3389/fpsyt.2021.731348 (PMC8585741; doi:10.3389/fpsyt.2021.731348)
Supplement: Supplementary file 1 [file Data_Sheet_1.PDF]

COVID-19'un Türkiye'deki Üniversite Öğrencilerinin Kaygı ve Stres Düzeyleri Üzerine Etkisi

Sayın Gönüllü,

Sizi, "COVID-19'un Türkiye'deki Üniversite Öğrencilerinin Kaygı ve Stres Düzeyleri Üzerine Etkisi" başlıklı araştırmamıza katılmaya davet ediyoruz. Bu araştırmanın amacı, COVID-19 hastalığının Türkiye'deki üniversite öğrencilerinin kaygı ve stres düzeylerini nasıl etkilediğini en gerçekçi şekilde ortaya koyarak öğrencilerin uzun dönemde yaşayacağı olası problemlere karşı önlemler alınması konusunda farkındalık yaratmaktır.

Cevaplayacağınız anket 3 bölümden oluşmaktadır. Bu kapsamda sizlerin genel bilgileri, COVİD-19'a bağl kaygı ve stres durumları araştırılacaktır. Anketin tamamını yanıtlamanız için gereken süre yaklaşık 5 dakikadır.

Araştırmamıza katılım gönüllülük esasına bağlıdır. Araştırmada yer almayı reddedebilirsiniz ya da başladıktan sonra yarıda bırakabilirsiniz. Cevaplamak istemediğiniz soruları boş bırakabilirsiniz. Bu çalışma sizler için herhangi bir risk faktörü içermemektedir. Anketi doldururken sizden kimlik bilgileriniz talep edilmeyecektir. Araştırmadan çekilmeniz halinde, sizle ilgili veriler araştırma dahilinde kullanılmayacaktır. Anket kapsamında paylaşmış olduğunuz bilgiler, ilgili araştırmacılar haricinde, hiçbir şekilde üçüncü şahıslarla paylaşılmayacaktır. Bu araştırmada herhangi bir kâr amacı güdülmemektedir. Bu araştırmanın sonuçları bilimsel amaçlarla kullanılacaktır. Bu anketi yanıtlamaya başlamış olmanız, araştırmaya katılmak için onay verdiğiniz anlamında kabul edilecektir.

*Required

Demografik Bilgiler

Bu bölüm katılımcıların genel bilgilerini öğrenmeye yönelik sorulardan oluşmaktadır. Burada toplamda 16 soru yer almaktadır. Cevaplamaya istediğiniz yerden başlayabilirsiniz.

1. Kaç yaşındasınız?

Lütfen sayısal olarak belirtiniz.

____

2. Cinsiyetiniz nedir?

Mark only one oval.

- Kadın
- Erkek
- Other: ______________________

3. Sigara kullanıyor musunuz?

Mark only one oval.

- Evet
- Hayır

4. Bir önceki soruya "Evet" yanıtını verdiyseniz, lütfen günlük miktarını adet veya paket olarak belirtiniz.

____

5. Alkol kullanıyor musunuz?

CDC tarafından önerilen günlük alkol alım miktarı kadınlarda 1 kadeh, erkeklerde ise 2 kadehtir.

Mark only one oval.

- Sıklıkla
- Sosyal içici
- Kullanmıyor

6. Kronik bir rahatsızlığınız var mı?

Mark only one oval.

- Evet
- Hayır

7. Bir önceki soruda "Evet" yanıtını verdiyseniz rahatsızlığınızı kısaca belirtebilirsiniz.

8. Düzenli olarak kullandığınız bir ilacınız var mı?

Mark only one oval.

- Evet
- Hayır

9. Bir önceki soruda "Evet" yanıtını verdiyseniz kullandığınız ilaçları belirtebilirsiniz.

10. Geçmişte / şu an psikolojik bir rahatsızlığınız sebebiyle yardım aldınız mı / alıyor musunuz?

Mark only one oval.

- Evet
- Hayır

11. Bir önceki soruda "Evet" yanıtını verdiyseniz rahatsızlığınızı kısaca belirtebilirsiniz.

12. Hangi üniversitede okuyorsunuz?

13. Okuduğunuz üniversitede güncel olarak hangi bölümde / alanda eğitim almaktasınız?

14. Güncel olarak öğrenim gördüğünüz yükseköğretim düzeyi nedir?

Mark only one oval.

- Ön lisans
- Lisans
- Yüksek lisans
- Doktora
- Tıp
- Diş hekimliği
- Other: ____________________

15. Öğreniminizin kaçıncı yılındasınız?

Mark only one oval.

- Hazırlık
- 1
- 2
- 3
- 4
- 5
- 6
- Other: ____________________

16. Pandemi süresince okuduğunuz bölümle / alanla ilgili sağlık kuruluşlarında (Ör. Hastane) uygulamalı eğitim almanız gerekti mi?

Mark only one oval.

- Evet
- Hayır

17. Üniversite eğitimi almak için düzenli olarak yaşadığınız şehirden ayrılmanız gerekiyor mu / okulunuz düzenli yaşadığınız şehirden farklı bir şehirde mi?

Mark only one oval.

- Evet
- Hayır

18. Pandemi öncesinde üniversite eğitimi aldığınız şehirde aileniz ile mi yaşıyordunuz yoksa ay mı?

Mark only one oval.

- Aile ile birlikte
- Aileden ayrı

19. Pandemi başladıktan sonra üniversite eğitimi aldığınız şehirde aileniz ile mi yaşıyorsunuz yoksa ayrı mı?

Mark only one oval.

- Aile ile birlikte
- Aileden ayrı

20. Ailenizde / akrabalarınızda COVİD-19 hastalığını geçirmiş / atlatmış herhangi birisi bulunuy mu?

Mark only one oval.

- Evet
- Hayır

21. Ailenizden / akrabalarınızdan birini COVİD-19 sebebiyle kaybettiniz mi?

Mark only one oval.

- Evet
- Hayır

COVID-19 Anksiyete Ölçeği

Bu bölümde katılımcıların COVİD-19 hastalığına bağlı kaygı (anksiyete) durumları araştırılacaktır. Anketin bu kısmı toplamda 5 sorudan oluşmaktadır. Cevaplamaya istediğiniz sorudan başlayabilirsiniz.

22. 1. Koronavirus ile ilgili haberleri okuduğum veya dinlediğim zaman başımın donduğunu ve sersemleştiğimi hissettim veya bayılacakmış gibi oldum. *

1 - Hiç, 2 - Nadir (1 veya 2 günden az), 3 - Birkaç gün, 4 - 7 günden fazla, 5 - Son 2 hafta boyunca neredeyse her gün

Mark only one oval.

	1	2	3	4	5	
Hiç						Son 2 hafta boyunca neredeyse her gün

23. 2. Koronavirüsü düşündüğüm için uykuya dalmada ya da uyumada sorun yaşadım. *

1 - Hiç, 2 - Nadir (1 veya 2 günden az), 3 - Birkaç gün, 4 - 7 günden fazla, 5 - Son 2 hafta boyunca neredeyse her gün

Mark only one oval.

	1	2	3	4	5	
Hiç						Son 2 hafta boyunca neredeyse her gün

24. 3. Koronavirüs ile ilgili konuları düşündüğümde ya da bu konulara maruz kaldığımda inme inmiş gibi hissettim veya donup kaldım. *

1 - Hiç, 2 - Nadir (1 veya 2 günden az), 3 - Birkaç gün, 4 - 7 günden fazla, 5 - Son 2 hafta boyunca neredeyse her gün

Mark only one oval.

	1	2	3	4	5	
Hiç	◯	◯	◯	◯	◯	Son 2 hafta boyunca neredeyse her gün

25. 4. Koronavirüs ile ilgili konuları düşündüğümde ya da bu konulara maruz kaldığımda iştahır kaçtı. *

1 - Hiç, 2 - Nadir (1 veya 2 günden az), 3 - Birkaç gün, 4 - 7 günden fazla, 5 - Son 2 hafta boyunca neredeyse her gün

Mark only one oval.

	1	2	3	4	5	
Hiç	◯	◯	◯	◯	◯	Son 2 hafta boyunca neredeyse her gün

26. 5. Koronavirüs ile ilgili konuları düşündüğümde ya da bu konulara maruz kaldığımda mide bulantısı ya da mide problemleri yaşadım. *

1 - Hiç, 2 - Nadir (1 veya 2 günden az), 3 - Birkaç gün, 4 - 7 günden fazla, 5 - Son 2 hafta boyunca neredeyse her gün

Mark only one oval.

	1	2	3	4	5	
Hiç	◯	◯	◯	◯	◯	Son 2 hafta boyunca neredeyse her gün

COVID-19 Stres Ölçeği

Bu bölümde katılımcıların COVİD-19 hastalığına bağlı stres durumları araştırılacaktır. Anketin bu kısm toplamda 36 sorudan oluşmaktadır. Cevaplamaya istediğiniz sorudan başlayabilirsiniz.

27. 1. Virüse yakalanmaktan endişelenirim. *

1 - Hiçbir zaman, 2 - Çok nadir, 3 - Bazen, 4 - Çoğu zaman, 5 - Her zaman

Mark only one oval.

	1	2	3	4	5	
Hiçbir zaman	⬭	⬭	⬭	⬭	⬭	Her zaman

28. 2. Ailemi virüsten koruyamayacağımdan endişelenirim. *

1 - Hiçbir zaman, 2 - Çok nadir, 3 - Bazen, 4 - Çoğu zaman, 5 - Her zaman

Mark only one oval.

	1	2	3	4	5	
Hiçbir zaman	⬭	⬭	⬭	⬭	⬭	Her zaman

29. 3. Hastalanmaları durumunda, sağlık sistemimizin sevdiklerimi koruyamayacağından endişelenirim. *

1 - Hiçbir zaman, 2 - Çok nadir, 3 - Bazen, 4 - Çoğu zaman, 5 - Her zaman

Mark only one oval.

	1	2	3	4	5	
Hiçbir zaman	⬭	⬭	⬭	⬭	⬭	Her zaman

30. 4. Hastalanmam durumunda, sağlık sistemimizin beni virüsten koruyamayacağından endişelenirim. *

1 - Hiçbir zaman, 2 - Çok nadir, 3 - Bazen, 4 - Çoğu zaman, 5 - Her zaman

Mark only one oval.

	1	2	3	4	5	
Hiçbir zaman						Her zaman

31. 5. Temel hijyen kurallarının (Ör. El yıkama) virüsten korunmam için yeterli olmadığından endişelenirim. *

1 - Hiçbir zaman, 2 - Çok nadir, 3 - Bazen, 4 - Çoğu zaman, 5 - Her zaman

Mark only one oval.

	1	2	3	4	5	
Hiçbir zaman						Her zaman

32. 6. Sosyal mesafenin beni virüsten korumak için yeterli olmadığından endişelenirim. *

1 - Hiçbir zaman, 2 - Çok nadir, 3 - Bazen, 4 - Çoğu zaman, 5 - Her zaman

Mark only one oval.

	1	2	3	4	5	
Hiçbir zaman						Her zaman

33. 7. Marketlerde yiyeceklerin bitmesinden endişelenirim. *

1 - Hiçbir zaman, 2 - Çok nadir, 3 - Bazen, 4 - Çoğu zaman, 5 - Her zaman

Mark only one oval.

	1	2	3	4	5	
Hiçbir zaman	⬭	⬭	⬭	⬭	⬭	Her zaman

34. 8. Marketlerin uzun bir süre kapanacağından endişelenirim. *

1 - Hiçbir zaman, 2 - Çok nadir, 3 - Bazen, 4 - Çoğu zaman, 5 - Her zaman

Mark only one oval.

	1	2	3	4	5	
Hiçbir zaman	⬭	⬭	⬭	⬭	⬭	Her zaman

35. 9. Marketlerde temizlik veya dezenfeksiyon malzemelerinin bitmesinden endişelenirim. *

1 - Hiçbir zaman, 2 - Çok nadir, 3 - Bazen, 4 - Çoğu zaman, 5 - Her zaman

Mark only one oval.

	1	2	3	4	5	
Hiçbir zaman	⬭	⬭	⬭	⬭	⬭	Her zaman

36. 10. Eczanelerde soğuk algınlığı veya grip ilaçlarının bitmesinden endişelenirim. *

1 - Hiçbir zaman, 2 - Çok nadir, 3 - Bazen, 4 - Çoğu zaman, 5 - Her zaman

Mark only one oval.

	1	2	3	4	5	
Hiçbir zaman	⬭	⬭	⬭	⬭	⬭	Her zaman

37. 11. Marketlerde suyun bitmesinden endişelenirim. *

1 - Hiçbir zaman, 2 - Çok nadir, 3 - Bazen, 4 - Çoğu zaman, 5 - Her zaman

Mark only one oval.

	1	2	3	4	5	
Hiçbir zaman	◯	◯	◯	◯	◯	Her zaman

38. 12. Eczanelerden reçeteli alınan özel ilaçların bitmesinden endişelenirim. *

1 - Hiçbir zaman, 2 - Çok nadir, 3 - Bazen, 4 - Çoğu zaman, 5 - Her zaman

Mark only one oval.

	1	2	3	4	5	
Hiçbir zaman	◯	◯	◯	◯	◯	Her zaman

39. 13. Yabancı uyruklu kişilerin ülkeme virüsü yaymasından endişelenirim. *

1 - Hiçbir zaman, 2 - Çok nadir, 3 - Bazen, 4 - Çoğu zaman, 5 - Her zaman

Mark only one oval.

	1	2	3	4	5	
Hiçbir zaman	◯	◯	◯	◯	◯	Her zaman

40. 14. Yabancı yiyeceklerde uzmanlaşmış bir restorana (Ör. Çin Lokantası) gitsem, virüse yakalanma konusunda endişelenirim. *

1 - Hiçbir zaman, 2 - Çok nadir, 3 - Bazen, 4 - Çoğu zaman, 5 - Her zaman

Mark only one oval.

	1	2	3	4	5	
Hiçbir zaman	◯	◯	◯	◯	◯	Her zaman

41. 15. Yabancı uyruklu kişiler ile temasa geçme konusunda endişelenirim, çünkü virüs taşıyor olabilirler. *

1 - Hiçbir zaman, 2 - Çok nadir, 3 - Bazen, 4 - Çoğu zaman, 5 - Her zaman

Mark only one oval.

	1	2	3	4	5	
Hiçbir zaman	◯	◯	◯	◯	◯	Her zaman

42. 16. Yabancı uyruklu biriyle tanışırsam, virüs bulaşmış olabileceğinden endişelenirim. *

1 - Hiçbir zaman, 2 - Çok nadir, 3 - Bazen, 4 - Çoğu zaman, 5 - Her zaman

Mark only one oval.

	1	2	3	4	5	
Hiçbir zaman	◯	◯	◯	◯	◯	Her zaman

43. 17. Bir grup yabancının bulunduğu bir asansördeysem, virüs bulaşmış olmasından endişelenirim. *

1 - Hiçbir zaman, 2 - Çok nadir, 3 - Bazen, 4 - Çoğu zaman, 5 - Her zaman

Mark only one oval.

	1	2	3	4	5	
Hiçbir zaman	◯	◯	◯	◯	◯	Her zaman

44. 18. Yabancıların virüsü yaydığından endişeliyim çünkü onlar bizim kadar temiz değiller. *

1 - Hiçbir zaman, 2 - Çok nadir, 3 - Bazen, 4 - Çoğu zaman, 5 - Her zaman

Mark only one oval.

	1	2	3	4	5	
Hiçbir zaman	◯	◯	◯	◯	◯	Her zaman

45. 19. Kamusal bir alanda (Ör. kapı kolu) bir şeye dokunduğumda virüsü yakalayacağımdan endişelenirim. *

1 - Hiçbir zaman, 2 - Çok nadir, 3 - Bazen, 4 - Çoğu zaman, 5 - Her zaman

Mark only one oval.

	1	2	3	4	5	
Hiçbir zaman	◯	◯	◯	◯	◯	Her zaman

46. 20. Biri yakınımda öksürür veya hapşırırsa, virüse yakalanacağımdan endişelenirim. *

1 - Hiçbir zaman, 2 - Çok nadir, 3 - Bazen, 4 - Çoğu zaman, 5 - Her zaman

Mark only one oval.

	1	2	3	4	5	
Hiçbir zaman	◯	◯	◯	◯	◯	Her zaman

47. 21. Etrafımdaki insanların bana virüs bulaştıracağından endişelenirim. *

1 - Hiçbir zaman, 2 - Çok nadir, 3 - Bazen, 4 - Çoğu zaman, 5 - Her zaman

Mark only one oval.

	1	2	3	4	5	
Hiçbir zaman	◯	◯	◯	◯	◯	Her zaman

48. 22. Nakit para işlemlerinde ve değişiminde virüs bulaşmasından endişelenirim. *

1 - Hiçbir zaman, 2 - Çok nadir, 3 - Bazen, 4 - Çoğu zaman, 5 - Her zaman

Mark only one oval.

	1	2	3	4	5	
Hiçbir zaman	◯	◯	◯	◯	◯	Her zaman

49. 23. Nakit para taşınmasında ve ATM'den çekilen paralardan dolayı virüse yakalanabileceğimden endişelenirim. *

1 - Hiçbir zaman, 2 - Çok nadir, 3 - Bazen, 4 - Çoğu zaman, 5 - Her zaman

Mark only one oval.

	1	2	3	4	5	
Hiçbir zaman	◯	◯	◯	◯	◯	Her zaman

50. 24 Kargolarıma, kuryeler tarafından virüs bulaştırıldığından endişelenirim. *

1 - Hiçbir zaman, 2 - Çok nadir, 3 - Bazen, 4 - Çoğu zaman, 5 - Her zaman

Mark only one oval.

	1	2	3	4	5	
Hiçbir zaman	◯	◯	◯	◯	◯	Her zaman

51. 25. Vücuduma virüs bulaştığını düşündüğümde, bir şeylere konsantre olmakta zorlanırım. *

1 - Hiçbir zaman, 2 - Çok nadir, 3 - Bazen, 4 - Çoğu zaman, 5 - Her zaman

Mark only one oval.

	1	2	3	4	5	
Hiçbir zaman	◯	◯	◯	◯	◯	Her zaman

52. 26. Virüsle ilgili daha önce gördüğüm rahatsız edici görüntüler, irademe rağmen aklıma gelmektedir. *

1 - Hiçbir zaman, 2 - Çok nadir, 3 - Bazen, 4 - Çoğu zaman, 5 - Her zaman

Mark only one oval.

	1	2	3	4	5	
Hiçbir zaman	◯	◯	◯	◯	◯	Her zaman

53. 27. Virüs hakkında endişelendiğimde uyku sorunları yaşamaktayım. *

1 - Hiçbir zaman, 2 - Çok nadir, 3 - Bazen, 4 - Çoğu zaman, 5 - Her zaman

Mark only one oval.

	1	2	3	4	5	
Hiçbir zaman	◯	◯	◯	◯	◯	Her zaman

54. 28. Virüs istemediğim halde bir şekilde sürekli aklıma gelmektedir. *

1 - Hiçbir zaman, 2 - Çok nadir, 3 - Bazen, 4 - Çoğu zaman, 5 - Her zaman

Mark only one oval.

	1	2	3	4	5	
Hiçbir zaman	◯	◯	◯	◯	◯	Her zaman

55. 29. Virüsün aklıma geldiğinde terleme, nefes alışında değişim ve hızlı kalp çırpıntısı gibi fiziksel reaksiyonlar yaşamaktayım. *

1 - Hiçbir zaman, 2 - Çok nadir, 3 - Bazen, 4 - Çoğu zaman, 5 - Her zaman

Mark only one oval.

	1	2	3	4	5	
Hiçbir zaman	◯	◯	◯	◯	◯	Her zaman

56. 30. Virüs hakkında kötü rüyalar gördüm. *

1 - Hiçbir zaman, 2 - Çok nadir, 3 - Bazen, 4 - Çoğu zaman, 5 - Her zaman

Mark only one oval.

	1	2	3	4	5	
Hiçbir zaman	◯	◯	◯	◯	◯	Her zaman

57. 31. İnternette COVID-19 tedavilerini hakkında aramalar yaptım. *

1 - Hiçbir zaman, 2 - Çok nadir, 3 - Bazen, 4 - Çoğu zaman, 5 - Her zaman

Mark only one oval.

	1	2	3	4	5	
Hiçbir zaman	◯	◯	◯	◯	◯	Her zaman

58. 32. Sağlık uzmanlarından (Ör. Doktorlar veya eczacılar) COVID-19 hakkında bilgi talep ettir *

1 - Hiçbir zaman, 2 - Çok nadir, 3 - Bazen, 4 - Çoğu zaman, 5 - Her zaman

Mark only one oval.

	1	2	3	4	5	
Hiçbir zaman	◯	◯	◯	◯	◯	Her zaman

59. 33. COVID-19 hakkında YouTube videoları izledim. *

1 - Hiçbir zaman, 2 - Çok nadir, 3 - Bazen, 4 - Çoğu zaman, 5 - Her zaman

Mark only one oval.

	1	2	3	4	5	
Hiçbir zaman	◯	◯	◯	◯	◯	Her zaman

60. 34. Vücudumu enfeksiyon belirtileri açısından kontrol ederim (Ör. Vücut sıcaklığı ölçümü).

1 - Hiçbir zaman, 2 - Çok nadir, 3 - Bazen, 4 - Çoğu zaman, 5 - Her zaman

Mark only one oval.

	1	2	3	4	5	
Hiçbir zaman	◯	◯	◯	◯	◯	Her zaman

61. 35. COVID-19 hastalığına yakalanma ve tedavisi konusunda arkadaşlarımdan veya ailemde görüş istedim. *

1 - Hiçbir zaman, 2 - Çok nadir, 3 - Bazen, 4 - Çoğu zaman, 5 - Her zaman

Mark only one oval.

	1	2	3	4	5	
Hiçbir zaman	⬭	⬭	⬭	⬭	⬭	Her zaman

62. 36. COVID-19 ile ilgili sosyal medya gönderileri paylaştım. *

1 - Hiçbir zaman, 2 - Çok nadir, 3 - Bazen, 4 - Çoğu zaman, 5 - Her zaman

Mark only one oval.

	1	2	3	4	5	
Hiçbir zaman	⬭	⬭	⬭	⬭	⬭	Her zaman

This content is neither created nor endorsed by Google.

Google Forms
